# Simultaneous X-ray Video-Fluoroscopy and Pulsed Ultrasound Velocimetry Analyses of the Pharyngeal Phase of Swallowing of Boluses with Different Rheological Properties

**DOI:** 10.1007/s00455-020-10092-4

**Published:** 2020-02-11

**Authors:** Waqas M. Qazi, Olle Ekberg, Johan Wiklund, Rashid Mansoor, Mats Stading

**Affiliations:** 1 Agrifood and Bioscience, Product Design and Perception, RISE, Research Institutes of Sweden AB, Göteborg, Sweden; 2 Department of Industrial and Material Sciences, Chalmers University of Technology, Göteborg, Sweden; 3 Diagnostic Centre of Imaging and Functional Medicine, Skåne University Hospital, Lund, Sweden; 4 Incipientus Ultrasound Flow Technologies AB, Frans Perssons Väg 6, 412 76 Göteborg, Sweden; 5 Nuffield Department of Medicine, Centre for Tropical Medicine and Global Health, University of Oxford, Oxford, UK

**Keywords:** Deglutition, Deglutition disorders, Rheology, Ultrasound, Video-fluoroscopy, Fluids

## Abstract

The Ultrasound Velocity Profiling (UVP) technique allows real-time, non-invasive flow mapping of a fluid along a 1D-measuring line. This study explores the possibility of using the UVP technique and X-ray video-fluoroscopy (XVF) to elucidate the deglutition process with the focus on bolus rheology. By positioning the UVP probe so that the pulsed ultrasonic beam passes behind the air-filled trachea, the bolus flow in the pharynx can be measured. Healthy subjects in a clinical study swallowed fluids with different rheological properties: Newtonian (constant shear viscosity and non-elastic); Boger (constant shear viscosity and elastic); and shear thinning (shear rate-dependent shear viscosity and elastic). The results from both the UVP and XVF reveal higher velocities for the shear thinning fluid, followed by the Boger and the Newtonian fluids, demonstrating that the UVP method has equivalent sensitivities for detecting the velocities of fluids with different rheological properties. The velocity of the contraction wave that clears the pharynx was measured in the UVP and found to be independent of bolus rheology. The results show that UVP not only assesses accurately the fluid velocity in a bolus flow, but it can also monitor the structural changes that take place in response to a bolus flow, with the added advantage of being a completely non-invasive technique that does not require the introduction of contrast media.

**Electronic supplementary material:**

The online version of this article (10.1007/s00455-020-10092-4) contains supplementary material, which is available to authorized users.

## Introduction

The rheological properties of the bolus profoundly influence the swallowing process in normal individuals and those suffering from dysphagia, i.e. abnormal swallowing [1, 2]. People with normal swallowing function adjust to the bolus rheology, which is a manifestation of the internal resistance to flow [3]. As low-viscosity fluids deform and flow with a higher velocity during swallowing, they may enter into the larynx if the laryngeal closure does not occur in a timely fashion [4, 5]. The rapid transport of the bolus during the pharyngeal phase of swallowing is challenging to handle for patients who are suffering from dysphagia [5]. It is widely accepted that the closure response of the airways is too slow to react adequately to the rapid flows of thinner liquids [6].

Patients who are suffering from dysphagia are restricted to consuming thickened fluids, which is a well-established food-based strategy for the management of dysphagia [7]. The fluids that are served to these patients are thickened with commercially available powdered thickeners [8]. The rationale for adding the thickeners is based on the concept of increasing bolus viscosity, thereby decreasing bolus velocity during swallowing [4, 9]. This enables individuals who are suffering from dysphagia to perform the necessary muscular adjustment to the fluids being swallowed. However, a bolus of high viscosity requires the application of an additional force to push it through the oropharyngeal apparatus. Moreover, a bolus of high consistency that cannot be handled by the abnormal swallowing mechanism of the patients may lead to complications with food residues [10, 11]. Therefore, high consistency is not the only factor that promotes the safety of swallowing in individuals who are suffering from dysphagia.

Some studies [7, 12, 13] have suggested that a food bolus is subject to extensional deformation (elasticity), in addition to shear deformation, as it is compressed between the tongue and the palate. This results in the bolus being stretched without being disintegrated as it approaches the pharynx, due to it being more cohesive. In our previous study [6] performed on patients with swallowing disorders, we have shown that the patients responded better to the fluids that had elastic properties. Swallowing function with respect to fluid extensional viscosity has not been studied as intensively as fluid shear viscosity [12, 13].

Besides the technical issues associated with measuring extensional viscosity, it is very difficult to study and present the effect of pure elasticity. Most of the thickened fluids possess both shear and elastic components and, thus, are viscoelastic. Boger fluids (named after its inventor, David Boger, who introduced the concept in 1977) are fluids that possess high-level elasticity and have a constant shear viscosity, i.e. one that is independent of the shear rate [14]. The main difference between Newtonian and Boger fluids is that Boger fluids are highly elastic, while Newtonian fluids lack the elastic component. Therefore, pure elastic effects can be separated from pure viscous effects by comparing the results acquired using a Boger fluid and a Newtonian fluid simultaneously [15].

Examination of the swallowing function with respect to bolus rheology can be performed using ultrasound-based techniques, such as the Ultrasound Velocity Profiling (UVP). The UVP technique measures the velocity of a moving particle suspended in a fluid using the pulsed ultrasound velocity profiling methodology and the Doppler effect [16, 17]. To trace the velocity, the reflector must be moving with non-zero velocity either into or away from ultrasound beam axis. The shift in frequency is measured using Eq. (1):

1 $${fi}^{\text{Doppler}}=\frac{{2f}_{0}{V}_{i}{\text{cos}}\theta }{c}$$

where ${fi}^{\text{Doppler}}$ is the Doppler shift frequency, $c$ is the ultrasound velocity in a fluid, $fo$ is the frequency emitted by the ultrasound transducer, and $\mathrm{cos}\theta$ is the Doppler angle, i.e. the angle between the ultrasound beam and the moving particle.

Using the UVP technique, Hasegawa et al. [18] measured the velocities of yoghurt and water during pharyngeal transport. Water, which often causes aspiration during swallowing, travelled with a velocity of 0.5 m/s, while the velocity of yoghurt during pharyngeal transport was recorded as 0.2 m/s. Similarly, Gao and Kohyama [19] used the UVP technique to examine the influence of water volume on bolus kinematics. In that study, they concluded that reducing the volume of the water caused the passage time and the bolus velocity in the oesophagus to decrease.

To the best of our knowledge, no study has been performed to date in humans using a UVP technique that assesses the influence of rheology, and in particular fluid elasticity, on bolus kinematics in parallel with X-ray video-fluoroscopy (XVF). Thus, the aim of the present study was to determine, using simultaneous UVP and XVF, the velocities and structural changes that occur in response to the flows of boluses that have different rheological properties.

## Subjects and Methods

### Subjects

XVF study was conducted on three healthy subjects (1 female, 2 males), age range 34–56 years. All the subjects signed an informed consent form approved by the Swedish Ethical Review Authority. The approval number for the ethical review is Dnr 2019-00,435 (2018/1042).

### Fluids and Rheological Properties

The Newtonian fluid was based on Maltodextrin (Dextrose equivalent 12) from AVEBE Food (Veendam, The Netherlands), while the Boger fluid comprised 200 ppm of xanthan gum (Grindsted Xanthan Clear 80; Danisco France SAS, Melle, France) as the elasticity-providing polymer. The final viscosities of the Newtonian and Boger fluids were ~ 150 mPas. The commercial thickener Nutilis (Nutricia Nordic AB, Stockholm, Sweden) was included as the shear thinning fluid and was thickened to nectar-consistency according to the manufacturer’s guidelines.

The final viscosity was set to be within the nectar-thick consistency range (50–350 mPas) following the National Dysphagia Diet (NDD) scale. An appropriate amount of powder was added to adjust the viscosity to 150 mPas at a shear rate of 50 s^−1^, such that all the model fluids had the same viscosity of ~ 150 mPas at a shear rate of 50 s^−1^ (Table 1).

**Table 1:** Power-law parameters (*K* and *n*) of the given three fluids (mixed with the contrast media at a 1:1 ratio) and of the contrast media used in the current work

	Shear thinning	Boger	Newtonian	Contrast media
*K* (mPa.s)^n^	230.00 ± 0.00	240.00 ± 12.00	170.00 ± 53	60.00 ± 17.00
*n*	0.68 ± 0.08	0.88 ± 0.01	0.92 ± 0.07	0.91 ± 0.07

All values shown are means of three replicates ± SD, while the Power-law relation is $\eta ={K\dot{\gamma }}^{n-1}$. The viscosities of the three model fluids are ~ 150 mPas at a shear rate of 50 s^−1^, while the viscosity of the contrast media is ~ 37 mPas at 50 s^−1^

All values shown are means of three replicates ± SD, while the Power-law relation is $\eta ={K\dot{\gamma }}^{n-1}$. The viscosities of the three model fluids are ~ 150 mPas at a shear rate of 50 s^−1^, while the viscosity of the contrast media is ~ 37 mPas at 50 s^−1^.

To achieve radio-opacity, an iodinated contrast media, OMNIPAQUE™ (GE-Healthcare, Stockholm, Sweden) was mixed with the model fluids at a 1:1 ratio.

### Measurement of Shear Rheology

To measure the shear viscosity, an ARES-G2 rheometer (TA Instruments, New Castle, DE, USA) was used. The geometry used in the analysis was cone and plate. The diameter of the cone was 40 mm and the cone angle was 0.04°. The shear rate was varied within the range of 1–1000 s^−1^ while the measurements were made at room temperature (25 °C).

### Measurement of Extensional Rheology

The extensional viscosities of the thickened solutions were determined using the Hyperbolic Contraction Flow (HCF) system mounted on an Instron 5542 (Instron Corp., Canton, USA). The HCF method is described in detail elsewhere [19–21].

### X-ray Video-Fluoroscopic Examination

The XVF examination was performed in lateral projection and stored in the Picture Archiving and Communication System (PACS) of the x-ray instrument (*MultiDiagnost Eleva FD*; Philips, Eindhoven, The Netherlands). Recording at 16 frames per second (fps) was used. The SECTRA version IDS7 (Linköping, Sweden) PACS allowed calculations of the speed of movement of the bolus head and tail (the latter being equivalent to the contraction wave) through measurement of the distances that these structures (bolus head and tail) moved between frames.

### Ultrasound Velocity Profiling

**Fig. 1:**
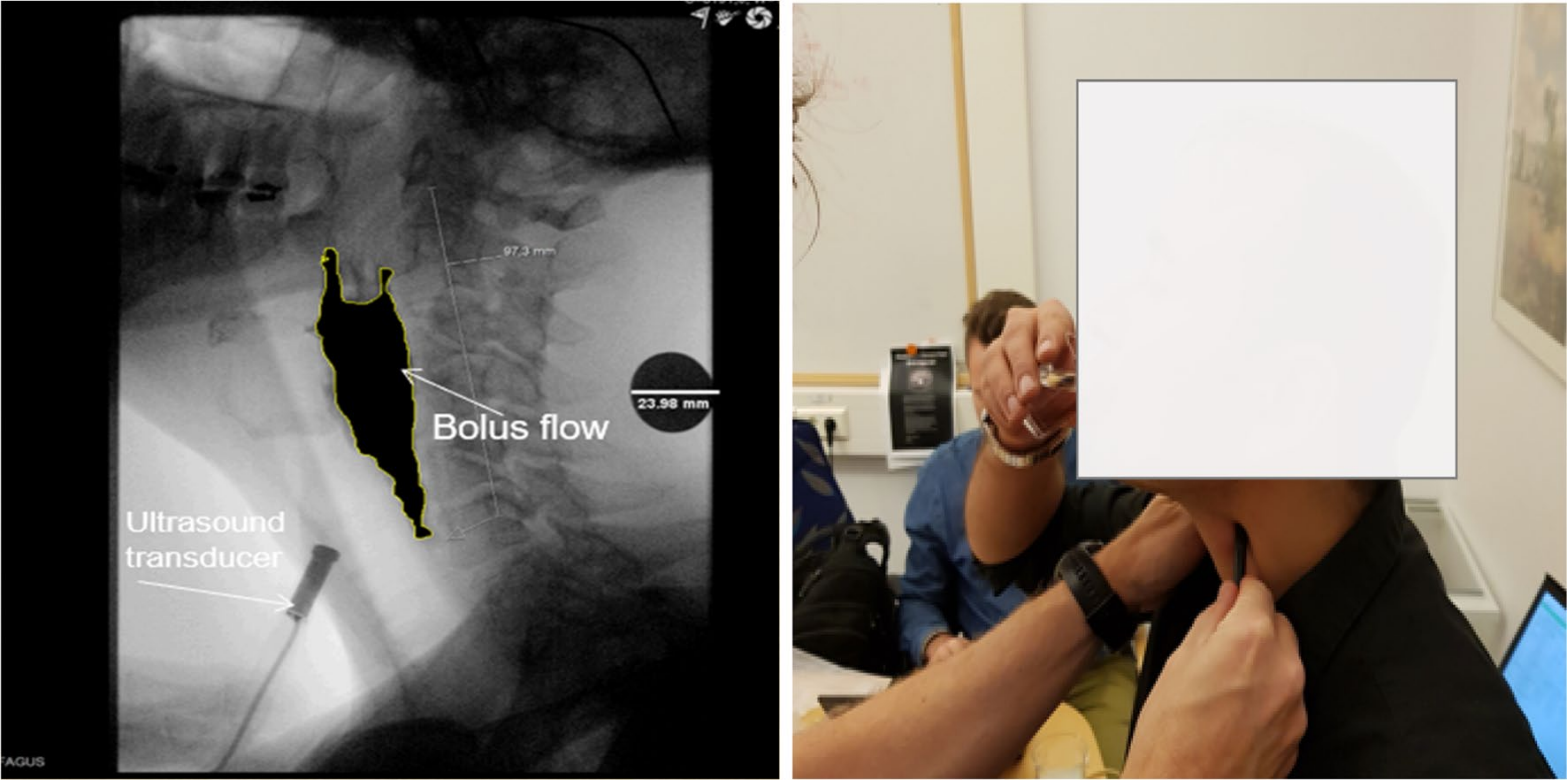
Image on the left is an example showing probe positioned at an angle and bolus flow during pharyngeal transport taken at the coronal plane. The scale bar (23.98 mm) in the image visible as a black circle is from a coin placed on the subject during the actual measurement. Image on the right shows the positioning of the transducer while measuring on the healthy subjects

In this work, an ultrasound (US) system from Incipientus Ultrasound Flow Technologies AB (Gothenburg, Sweden) was used. A 4-MHz US transducer based on a single active element was utilised. The frequency and transducer types were used based on their suitabilities to measure in relatively small, shallow organs such as the pharynx. The subjects sat in an upright position and the US probe was placed against the neck at a caudate angle of between ~ 30 and 40° to the posterior pharynx wall in the lateral projection (Fig. 1). The US probe was directed to the lower pharynx. The correct location in the pharynx for measuring the bolus flow and the angle of the measurement were determined in the presence of experienced practitioners. Determination of the Doppler angle was crucial to calculating the velocities in the pharynx.

In a previous study [3], the US pulse was directed at an upward angle of 60°. Similarly, in another study [18], the probe was manually placed at an angle of 35° against the necks of the subjects. In the present study, extra care was taken for the determination of the Doppler angle from the acquired video-fluoroscopy images (Fig. 1), as well as in the x-ray images using the SECTRA version IDS7 software, with confirmation using the open-source image processing software Image J. Thus, we were able to assess the variations in the determinations of the correct angle for individual swallows, thereby eliminating the human error. The angle determines the reported velocity as expressed in the direction of the bolus flow.

The subjects were instructed to hold in their mouth 15 ml of the pre-measured given fluid and to swallow the aliquot in a single go (temperature, 23 °C). Three sips of each fluid were taken by each subject.

### Analysis of the Bolus Head Velocity and Velocity of the Contraction Wave Following the Bolus and Pre-swallow Pharyngeal Movement

**Fig. 2:**
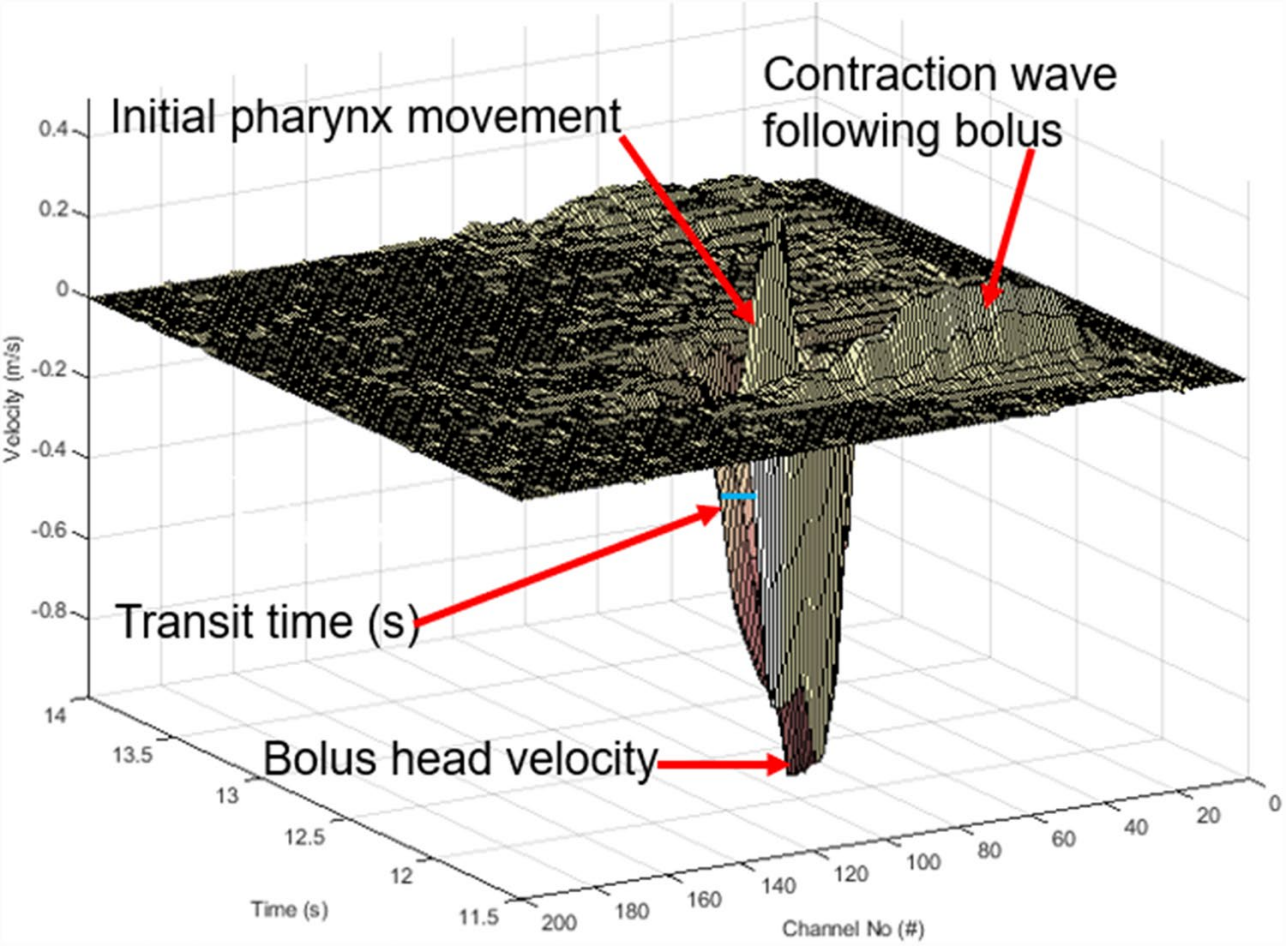
3D diagram demonstrating different movement recorded during pharyngeal bolus transport using a single-element transducer along a single measurement line. The movements recorded with the ultrasound transducer were in the order: initial pharynx movement, movement of the bolus head and the contraction wave following the bolus (in the form of series of small-amplitude oscillations). The velocity of the bolus head was measured in the direction away from the transducer

The measured velocity profiles were analysed for various parameters of clinical interest, such as the Bolus head velocity (the maximum velocity that the tip of the bolus achieves), the velocity of the contraction wave (the movement recorded by the US transducer as the main bolus transport takes place), and the initial movement (pre-swallow spike of the velocity of the pharynx prior to bolus entry into the pharynx), as shown in Fig. 2. The total number of velocity profiles differed for each subject due to the swallowing process being different for individuals. Thus, the required sampling rate was also different. It was possible to measure a maximum of 512 velocity profiles with the current measurement system.

The UVP instrument performs measurement along a pulsed beam axis, while the sampling data are recorded in multiple spatial positions called “gates” or “channels”.

The data presented in Fig. 2 are from a single-element transducer and were acquired along a single line over a short period of time. The initial upward movement of the pharynx prior to bolus arrival is visible in Fig. 2. The contraction wave is shown towards the end of the measurement as a series of small-amplitude oscillations in the data. The sampling rate for the US signals was100 MHz. However, the total time per profile was 47 ms.

### Statistical Methods

All statistical analyses were performed using the Stata MP 15 or Microsoft Excel 2010 software. Two outcomes were evaluated: (a) bolus tip velocity (m/s); and (b) contraction wave speed (mm/s). Univariable and multivariable linear regression models were fitted for outcomes to identify mean difference between the fluids and the techniques used.

## Results

### Extensional Rheology

**Fig. 3:**
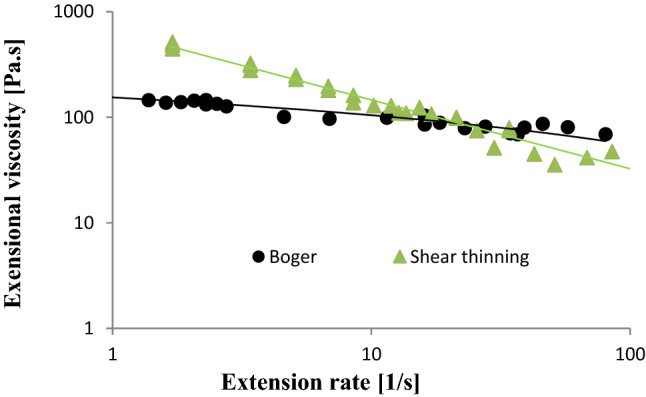
Extensional viscosity (expressed in Pa.s) of the fluids used in clinical examination

Fluid elasticity was expressed as the extensional viscosity determined by the HCF method (Fig. 3). The commercial thickener Nutilis fluid used in the clinical study that showed thinning behaviour in shear deformation also showed extension thinning behaviour, i.e. the extensional viscosity decreased with increasing extension rate, as found previously [6]. The Boger fluid was less-elastic than the shear thinning fluid at lower extension rates (< 10 s^−1^) and demonstrated less extension thinning than the shear thinning fluid (flow index: for Boger fluid, *n* = 0.81 and for shear thinning fluid, *n* = 0.35). The Newtonian fluid demonstrated no elasticity and therefore is not shown.

It is worth mentioning that although the shear thinning fluid showed higher elasticity at low extension rates, it is still not purely elastic but instead is viscoelastic and its influence on swallowing performance might reflect a shear thinning behaviour. Extensional rheology has been suggested by several groups as a factor that promotes safe swallowing [6, 7, 13, 22]. However, there are relatively few publications on this important material property with respect to safe swallowing. There is currently no study in the literature that takes the extension rate during swallowing into consideration. In the present study, the discussion on extensional rheology is restricted to whether or not the extensional viscosity can be detected using the UVP technique.

### Velocity of the Bolus Head

**Fig. 4:**
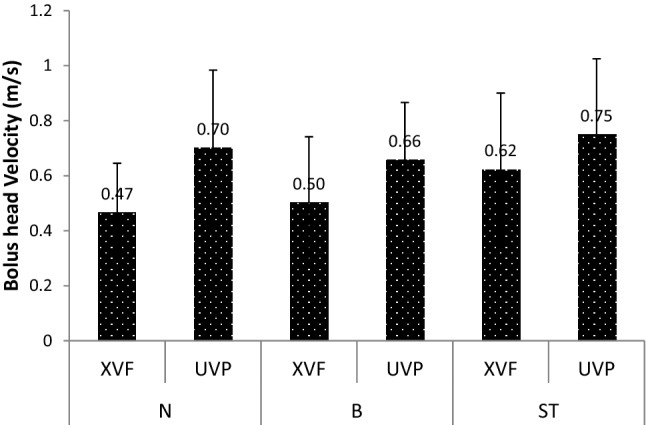
Measured velocities of the bolus head using XVF and UVP in the Newtonian (N), Boger (B), and shear thinning (ST) fluids. The given values are the average of three replicates acquired from three subjects

Figure 4 shows the velocities acquired using both the XVF and UVP techniques during pharyngeal bolus transport.

With the UVP technique, the highest velocities of the bolus head were recorded in the shear thinning fluid, followed by the Newtonian fluid and Boger fluid. However, these differences in the mean bolus head velocities were not statistically significant (Newtonian fluids compared to Boger fluids (*p* = 0.722); shear thinning fluids compared to Boger fluids (*p* = 0.462). With the XVF method, the velocities of the bolus head were highest for the shear thinning fluid, followed by the Boger and Newtonian fluid. However, no significant differences in the mean bolus head velocities between different fluids (Table 2; Supplementary Table S1) were recorded.

**Table 2:** Multivariable analysis with mean differences in velocity (m/s) and contraction wave speed for each fluid and technique

	Velocity (m/s)		Velocity of contraction wave speed (m/s)	
Parameter	Estimate (95% CI)	*p* value	Estimate (95% CI)	*p* value
Technique: UVP				
Newtonian fluids	0.043 (− 0.199 to 0.285)	0.722	0.013 (− 0.030 to 0.055)	0.554
Shear thinning fluids	0.092 (− 0.158 to 0.341)	0.462	0.006 (− 0.037 to 0.048)	0.786
Boger fluids	Reference		Reference	
Technique: XVF				
Newtonian fluid	− 0.036 (− 0.277 to 0.206)	0.768	0.009 (− 0.033 to 0.052)	0.665
Shear thinning fluid	0.119 (− 0.122 to 0.361)	0.325	0.008 (− 0.034 to 0.051)	0.696
Boger fluid	Reference		Reference	
Technique: XVF compared to UVP				
Newtonian fluid: XVF	− 0.234 (− 0.476 to 0.007)	0.057	− 0.037 (− 0.080 to 0.005)	0.083
UVP	Reference		Reference	
Shear thinning fluid: XVF	− 0.128 (− 0.378 to 0.121)	0.306	− 0.031 (− 0.074 to 0.011)	0.143
UVP	Reference		Reference	
Boger fluid: XVF	− 0.156 (− 0.397 to 0.086)	0.201	− 0.034 (− 0.076 to 0.009)	0.114
UVP	Reference		Reference	

*UVP* ultrasound velocity profiling, *XVF* X-ray video-fluoroscopy

Overall, for the three fluids examined, lower velocities were noted with the XVF technique compared to UVP. These differences were not statistically significant (Newtonian fluids: *p* = 0.057, shear thinning fluids: *p* = 0.306 and Boger fluids: *p* = 0.201).

The results indicate the UVP technique is as sensitive as or superior to XVP with respect to the measurement of velocities during pharyngeal bolus transport.

### Velocity of the Contraction Wave that Follows the Bolus

**Fig. 5:**
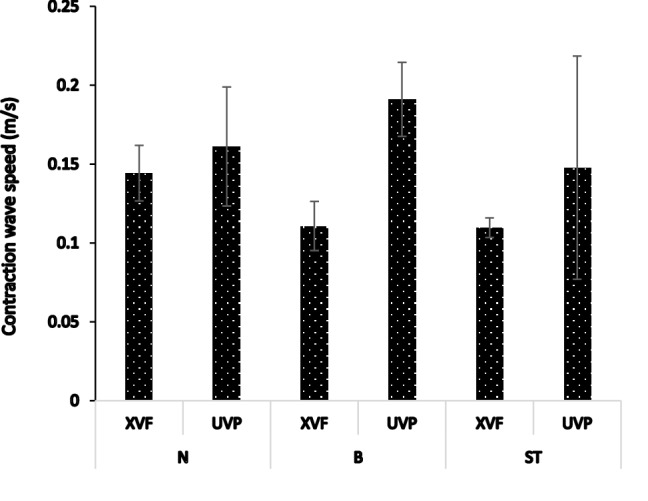
Velocities of the contraction waves measured in the subjects for the three different fluids using XVF and UVP. The values shown are the averages of three replicate measurements from three subjects. *N* Newtonian fluid, *B* Boger fluid, *ST* shear thinning fluid

The velocity of the contraction wave that clears the pharynx [23] was calculated using XVF and compared with the results acquired with the non-invasive UVP technique. The results of the UVP measurements suggested that this velocity is independent of the rheological properties of the fluids, as no statistically significant differences (Newtonian fluids compared Boger fluids, *p* = 0.554; shear thinning fluids compared Boger fluids, *p* = 0.786) were noted for the velocity of the contraction wave while performing measurement with the UVP. Similar observations were noted in the XVF technique (Newtonian fluids compared Boger fluids, *p* = 0.665; shear thinning fluids compared Boger fluids, *p* = 0.696) as shown in Fig. 5 and Table 2.

Furthermore, there was no significant difference in the measured mean velocities between the XVF and UVP methods for the different fluids (Newtonian fluid, *p* = 0.083; Boger fluid, *p* = 0.114 and shear thinning fluid, *p* = 0.143).

Overall, most of the results for the velocity of the contraction wave showed statistically non-significant differences with respect to the fluid rheological properties, as is expected for normal individuals.

## Discussion

In the UVP technique, slightly higher velocities were noted due to the higher resolution of this method compared to video-fluoroscopy analysis, which recorded the videos only at16 fps.

The shear thinning boluses travelled with higher velocities in most cases. As the viscosity of a shear thinning fluid decreases with increasing shear rate, it travels with a faster velocity. Thickened fluids always demonstrate shear thinning behaviour [5, 6, 24]. The Power-law rheological model describes this behaviour as:

$$\sigma =K (\dot{\gamma })^n\,{\text{or}}\,\eta = K\dot{\gamma }^{n - 1} ,$$

where σ, η, γ̇, *K*, and *n* are the shear stress, apparent viscosity, shear rate, consistency index, and flow index expressions, respectively. The degree of shear thinning is represented by the “*n*” value. Thus, a Newtonian fluid has *n* = 1, while *n* < 1 indicates the extent of shear thinning in the fluid. The shear rate increases substantially during pharyngeal bolus transport and especially in the lower part of the pharynx, as shown previously [13]. This explains why higher velocities were noted in the shear thinning samples with both of the techniques applied in the present work. The differences between the velocities of the different fluids were, however, not statistically significant due to many factors, including the use of normal subjects, a lower number of replications, and overall fewer differences in the viscosities of the tested model fluids. The differences in the flow index *n* between the different fluids were inevitably small (Newtonian fluid, *n* = 0.92 ± 0.07: Boger fluid, *n* = 0.88 ± 0.01; and shear thinning fluid, *n* = 0.68 ± 0.08), as the contrast media had to be mixed in appreciable amounts so as to achieve good visualisation during XVF.

In a Boger fluid, as opposed to the corresponding Newtonian counterpart, contraction is expected to yield a slower flow (due to the elastic component of the Boger fluid) as well as to contribute to the cohesiveness of the bolus and, thereby, safe swallowing. The elasticity could also have the advantage of avoiding post swallow residues. These arise from the extra effort required to push a bolus of high viscosity during pharyngeal transport that is known to create residues [25, 26]. In the current study, non-significant differences in the velocities were observed between the Boger and Newtonian fluid. This may be attributed to the healthy subjects’ ability to compensate for the different fluid rheologies, as discussed in detail in a previous study [27]. We speculate that the effect of elasticity is more pronounced in individuals who are suffering from dysphagia.

The overall velocities recorded here were higher than those reported previously (0.1–0.5 m/s by [3, 22, 28, 29]), due to the lower viscosities of the fluids used in the current study. The US probe used in this work was directed and the velocities were measured in the lower pharynx, as it was easy to measure in that region of the pharynx in this preliminary study with the settings obtained from the initial trial-and-error experiments. In the future, the velocities will be assessed throughout the entire pharyngeal region and using both the in vivo and in vitro analyses.

During pharyngeal swallowing, the main bolus flow is followed by the ridge-like contraction wave so as to clear the pharynx [30, 31]. The velocity of this contraction wave is almost constant (since it is under neural control from the brainstem), independent of the subjects tested, as proposed previously [32] in a study performed on ten healthy subjects who swallowed barium contrast media (60% w/v). In that study, it was hypothesised that the contraction wave velocity should be independent of the rheological properties of the fluid as the subjects that participated in the study were healthy. In the above-mentioned previous study [32], which was performed on healthy subjects, the velocity of the contraction wave varied within the range of 130–170 mm/s, which is similar to the velocity of the contraction wave recorded here by the UVP method, that is within the range of 148–191 mm/s. Due to the lower resolution (16 fps) of the video-fluoroscopy, lower velocities were recorded (range 110–133 mm/s).

Overall, the results show that for the three fluids examined, the UVP method recognises the independence of the contraction wave velocity as well if not better than XVF.

This work was initially aimed at investigating the possibility of using the non-invasive UVP technique for assessment of pharyngeal bolus transport, with comparison to XVF. Importantly, UVP can detect the initial pharyngeal movement, prior to bolus entry into the pharynx (not discussed in detail in this study). We found these initial preparatory movements to be very different in terms of fluid rheology, which probably reflects the ability of the body to compensate and adjust the swallowing effort in response to a specific fluid rheology. Furthermore, with the UVP system used in this work, the velocity distribution in the entire pharyngeal cavity can be measured, which allows calculation of the shear rate in the human pharynx. The shear rates for some individual swallows were > 1000 s^−1^ in the lower pharynx examined here. A discussion of the shear rate is avoided in this work as this was not the major aim of this study; a separate publication will discuss this aspect. Furthermore, it is planned that the present study will be extended to include patients who are suffering from dysphagia.

## Conclusion

In this work, we show that the UVP technique can be used to examine non-invasively the swallowing process, without the addition of contrast media. The UVP technique accurately detects and measures the velocities of fluids with different rheological properties. In addition, the structural changes that occur in the pharynx, such as the clearance wave following the bolus tail, were correctly detected by the UVP technique, as compared with the Gold standard method. Therefore, UVP in combination with XVF can be used to analyse the deglutition process in much more detail than is possible with XVF alone.

## Electronic supplementary material

Below is the link to the electronic supplementary material.

Electronic supplementary material 1 (DOCX 14 kb)
