# Supplementary material for: Simultaneous X-ray Video-Fluoroscopy and Pulsed Ultrasound Velocimetry Analyses of the Pharyngeal Phase of Swallowing of Boluses with Different Rheological Properties
Source: Dysphagia. 2020 Feb 11;35(6):898–906. doi: 10.1007/s00455-020-10092-4 (PMC7669789; doi:10.1007/s00455-020-10092-4)
Supplement: Supplementary file 1 — Electronic supplementary material 1 (DOCX 14 kb) [file 455_2020_10092_MOESM1_ESM.docx]

**Table** S1: Univariable analysis with mean difference in velocity, contraction wave speed and transit time for each fluid and technique

|  | Velocity (m/s) | | Velocity of contraction wave speed (m/s) | | | |
| --- | --- | --- | --- | --- | --- | --- |
| Parameter | Estimate (95% CI) | p-value | Estimate (95% CI) | | p-value | |
| Technique: UVP |  |  | |  | |  |
| Newtonian fluids | 0.043 (-0.219- 0.304) | 0.736 | 0.013 (-0.045- 0.07) | | 0.655 | |
| shear thinning fluids | 0.090 (-0.18- 0.36) | 0.496 | 0.006 (-0.052- 0.063) | | 0.838 | |
| Boger fluids | Reference |  | Reference | |  | |
| Technique: XVF |  |  | |  | |  |
| Newtonian fluids | -0.036 (-0.283- 0.212) | 0.768 | 0.009 (-0.013- 0.031) | | 0.403 | |
| shear thinning fluids | 0.119 (-0.128- 0.367) | 0.328 | 0.008 (-0.014- 0.031) | | 0.449 | |
| Boger fluids | Reference |  | Reference | |  | |
